# Severe Osteomalacia Related to Long-Term Intravenous Drug Abuse

**DOI:** 10.1177/2324709614548797

**Published:** 2014-09-02

**Authors:** Leslie Gamache, Mark R. Burge

**Affiliations:** 1 Health One, Denver Endocrinology, Denver, CO, USA; 2 University of New Mexico Health Sciences Center, Albuquerque, NM, USA

**Keywords:** osteomalacia, vitamin D, narcotic addiction

## Abstract

*Objective*. We present the clinical, biochemical, and imaging findings of a woman with vitamin D deficiency and severe osteomalacia related to intravenous heroin addiction. *Results*. A 54-year-old woman with a medical history significant for long-standing heroin abuse presented with complaints of bone pain, muscle cramping, and a left hip ulcer. She had been bed bound for approximately 1 year secondary to pain of uncertain etiology, and her husband was bringing her both food and drugs. She was admitted to the hospital for debridement of a right ischial ulcer. Further workup revealed osteomyelitis of the left hip and severe vitamin D deficiency. Radiologic evaluation demonstrated diffuse osteopenia with pseudofractures, as well as true fractures. *Conclusion*. This is the first case reported in the English literature of advanced osteomalacia resulting from a debilitating narcotic dependency. Vitamin D deficiency should be considered in patients with poor nutrition and prolonged sunlight deprivation from any cause.

## Introduction

Vitamin D is necessary to maintain bone integrity. A deficiency of vitamin D disturbs calcium homeostasis and causes a secondary hyperparathyroidism. This disruption can cause a spectrum of disease ranging from high bone turnover and decreased bone mass to impaired mineralization and osteomalacia.^1^ It had been assumed that the public health effort to fortify foods with vitamin D had eliminated vitamin D deficiency as a serious health problem. However, several recent publications have demonstrated increasing numbers of adults with low vitamin D levels.^2-4^ This hypovitaminosis likely contributes to fracture risk, but severe osteomalacia is still rare in the United States.

## Case Summary

### History

A 54-year-old Hispanic woman presented to the emergency room with complaints of bone pain, muscle cramping and a left hip ulcer. Her past medical history was significant for 30 years of intravenous heroin abuse. She had been bed bound for approximately 1 year secondary to pain of uncertain etiology. Her husband enabled her house-bound condition by bringing food and drugs to her. Review of systems was negative for symptoms of malabsorption, such as diarrhea or floating stools. She denied any falls or trauma. The patient had not had a menstrual period for more than 20 years, but claimed that she had 2 previous pregnancies with spontaneous abortions in her 30s. On physical examination, she was frail and ill appearing, but not in acute distress. She had contractures of the upper and lower extremities, as well as a 7 × 7 cm ulceration on the left ischium with serosanguinous drainage that she attributed to an old burn injury and that she admitted using for heroin injection. She was admitted for debridement of the ischial ulcer and for further workup of her bone pain.

### Laboratory Studies

Laboratory testing revealed classic biochemical findings of vitamin D deficiency, including 25-(OH)D_3_ < 5 ng/mL (reference range, 20-57 ng/mL), calcium 7.3 mg/dL (reference range 8.4-10.4 mg/dL), parathyroid hormone 1187 pg/mL (reference range, 10-65 pg/mL), 1,25-(OH)_2_D_3_ 17 pg/mL (reference range 15-75 pg/mL), phosphorus 2.1 mg/dL (reference range 2.3-5.6 mg/dL), and magnesium 2.0 meq/L (reference range 1.4-2.6 meq/L). Erythrocyte sedimentation rate was 35 mm/h (reference range 0-39 mm/h). Liver function tests showed aspartate transaminase 38 U/L (reference range 3-70 U/L), alanine transaminase 3 U/L (reference range 3-78 U/L), total protein 7.3 g/dl (reference range 5.9-8.3 g/dL), albumin 2.5 g/dL (reference range 3.1-4.7 g/dL), alkaline phosphatase 317 U/L (reference range 20-145 U/L), and total bilirubin 0.7 mg/dL (reference range 0.0-1.4 mg/dL). Prealbumin was 12.1 mg/dL (reference range 18-45 mg/dL). Thyrotropin was within normal limits at 1.66 µIU/mL (reference range 0.4-4.5 µIU/mL). Estrogen levels were not determined, but gonadotropins were suppressed, with luteinizing hormone <0.2 mIU/mL (reference range 1.1-11.6 mIU/mL for follicular phase) and follicle-stimulating hormone <1.0 mIU/mL (reference range 2.8-11.3 mIU/mL for follicular phase).

### Imaging Findings

**Figure 1.**
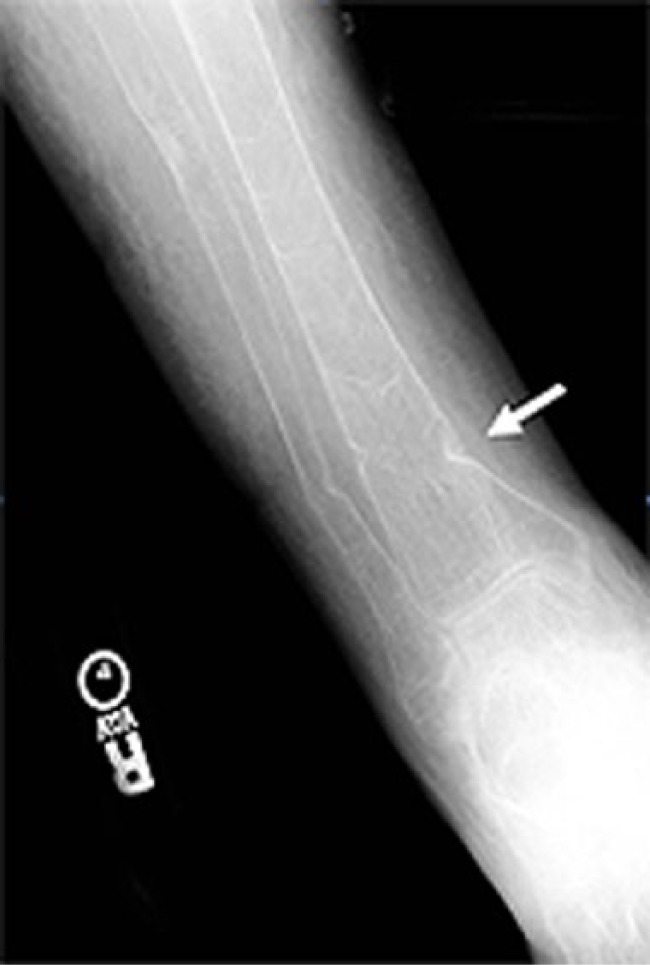
An anteroposterior radiograph of the right tibia and fibula shows striking osteopenia of both bones. Several pseudofractures (or “Looser zones”) are present, the most prominent of which is the buckle-type fracture at the distal diaphysis of the tibia (arrow).

**Figure 2.**
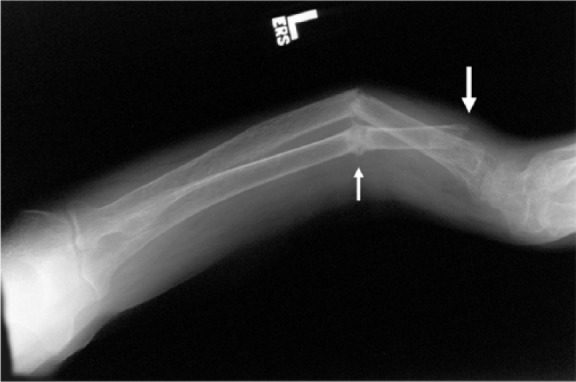
A radiograph of the left lateral forearm also shows marked bony demineralization. There are fractures at various stages of healing, as evidenced by callous formation at an old fracture (small arrow) and by sharp margins at an acute fracture (large arrow).

**Figure 3.**
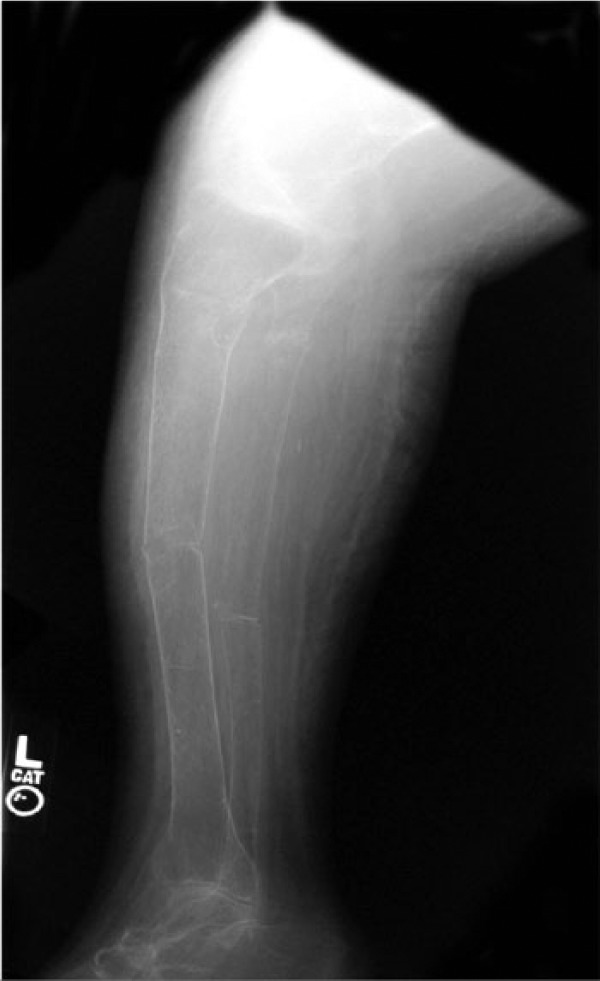
The lateral view of the left tibia and fibula shows severely osteopenic and gracile bones. Fractures involving the mid-tibial and mid-fibular shafts are also seen, but age assessment of these injuries is compromised because of the severe osteopenia.

**Figure 4.**
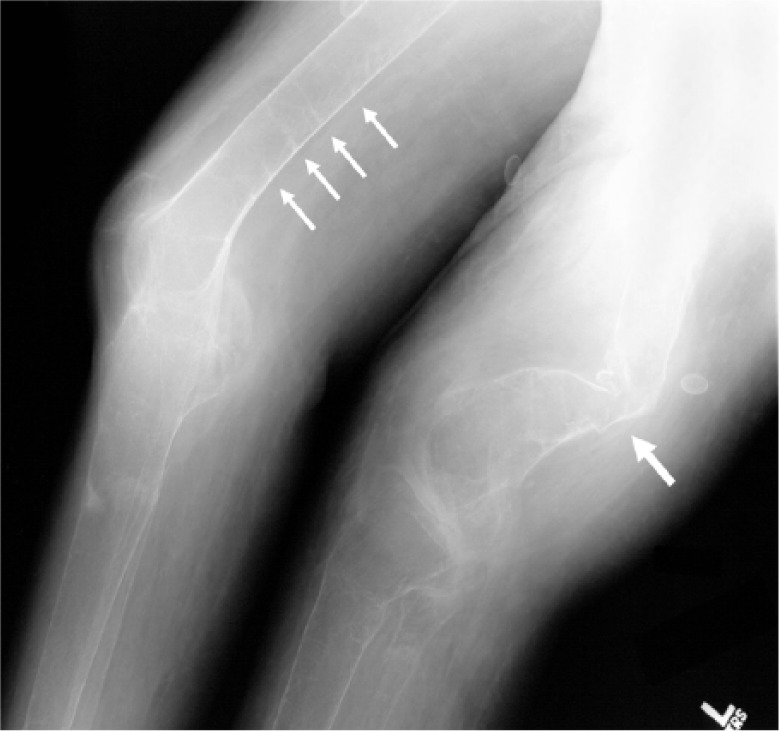
A radiograph of the distal femurs shows further evidence of badly malformed bones secondary to severe osteomalacia (large arrow), as well as several additional pseodofractures (small arrows).

A series of radiographs demonstrated the dramatic bone changes of osteopenia and osteomalacia. An anteroposterior radiograph of the right tibia and fibula (Figure 1) shows striking osteopenia of both bones. Several pseudofractures (also known as “Looser zones”) are present, the most prominent of which is the buckle-type fracture at the distal diaphysis of the tibia. A radiograph of the left lateral forearm (Figure 2) also showed marked bony demineralization. There were fractures at various stages of healing as evidenced by callous formation at an old fracture site and by sharp margins at an acute fracture. A lateral view of the left tibia and fibula (Figure 3) again show severely osteopenic and gracile bones. The outline of the fibula is barely visible because of the lack of cortical bone. Fractures involving the mid-tibial and mid-fibular shafts are also seen, but age assessment of these injuries is compromised because of the severe osteopenia. A radiograph of the distal femurs (Figure 4) shows badly misshapen bone and several additional pseudofractures attributable to severe osteomalacia.

## Discussion

Severe osteomalacia is seen uncommonly in current medical practice. Our case therefore provides a unique opportunity to view the biochemical and imaging findings of this condition. Osteomalacia results from inadequate mineralization of newly formed osteoid (bone protein matrix) in mature bones. As demonstrated in this case, radiographic findings include generalized osteopenia and pseudofractures. Pseudofractures are lucent areas oriented at right angles to the cortex and that incompletely span the diameter of the bone. These zones are considered a type of stress fracture, although true fractures can occur through the weakened areas.^5^

There are numerous causes of osteomalacia, but vitamin D deficiency is the most common cause. In our patient, the constellation of a low-normal serum calcium, low serum phosphorus, elevated alkaline phosphatase, increased parathyroid hormone and low 25-(OH)D_3_ are all typical of severe vitamin D deficiency. Although the workup was generally thorough, there are some tests that were not ordered that may have further elucidated this case, including 24-hour urinary calcium excretion, as well as markers of bone turnover, including bone formation markers such as bone specific alkaline phosphatase and osteocalcin, and bone resorption markers such as carboxy-terminal propeptide of type I collagen, urinary total pyridinoline and deoxypyridinoline, type I collagen cross-linked N-telopeptide, and type I collagen cross-linked C-telopeptide.^6^ This case was also complicated by acute osteomyelitis of the left ischium (with *Staphylococcus aureus*) and probable osteoporosis attributable to both hypogonadism and immobility.

Vitamin D is unique in that the human body relies on both endogenous production and exogenous sources to meet biologic requirements. This complex metabolism requires exposure to ultraviolet light and an appropriate diet.^7-9^ In our patient, a debilitating intravenous heroin addiction prevented both the necessary sunlight exposure and the adequate nutrition, as evidenced by her low prealbumin level. This case is further complicated by prolonged immobility, which has been well documented to exert deleterious wasting effects on the human skeleton.^10,11^ It could also be speculated that the narcotics had some previously undescribed, direct effect on bone demineralization. The multiple fractures observed at various stages of healing also raise the suspicion of domestic abuse, but the patient denied any trauma. During the hospital stay, a fracture occurred during a routine bed transfer, which lent support to the nontraumatic nature of the bone findings.

## Conclusion

This is the first reported case of advanced osteomalacia attributable to intravenous heroin addiction. Vitamin D deficiency and osteomalacia should be considered in all patients with poor nutrition and prolonged sunlight deprivation from any cause.
